# Phytotoxic impact of bifunctionalized silver nanoparticles (AgNPs-Cit-L-Cys) and silver nitrate (AgNO_3_) on chronically exposed callus cultures of *Populus nigra* L.

**DOI:** 10.1007/s11356-023-30690-7

**Published:** 2023-11-01

**Authors:** Valentina Iori, Valerio Giorgio Muzzini, Iole Venditti, Barbara Casentini, Maria Adelaide Iannelli

**Affiliations:** 1 Institute of Agricultural Biology and Biotechnology - National Research Council (IBBA-CNR), Strada Provinciale 35d, 9, 00010 Montelibretti, Rome, Italy; 2 Research Institute On Terrestrial Ecosystems - National Research Council (IRET-CNR), Strada Provinciale 35d, 9, 00010 Montelibretti, Rome, Italy; 3 Department of Sciences, University of Roma Tre, Via Della Vasca Navale 79, 00146 Rome, Italy; 4 Water Research Institute - National Research Council (IRSA-CNR), Strada Provinciale 35d, 9, 00010 Montelibretti, Rome, Italy

**Keywords:** Bifunctionalized nanosilver, Poplar, In vitro culture, Oxidative stress, Phytotoxicity, Ecosafety

## Abstract

Owing to the unique physicochemical properties and the low manufacturing costs, silver nanoparticles (AgNPs) have gained growing interest and their application has expanded considerably in industrial and agricultural sectors. The large-scale production of these nanoparticles inevitably entails their direct or indirect release into the environment, raising some concerns about their hazardous aspects. Callus culture represents an important tool in toxicological studies to evaluate the impact of nanomaterials on plants and their potential environmental risk. In this study, we investigated the chronic phytotoxic effects of different concentrations of novel bifunctionalized silver nanoparticles (AgNPs-Cit-L-Cys) and silver nitrate (AgNO_3_) on callus culture of *Populus nigra* L., a pioneer tree species in the riparian ecosystem. Our results showed that AgNPs-Cit-L-Cys were more toxic on poplar calli compared to AgNO_3_, especially at low concentration (2.5 mg/L), leading to a significant reduction in biomass production, accompanied by a decrease in protein content, a significant increase in both lipid peroxidation level, ascorbate peroxidase (APX), and catalase (CAT) enzymatic activities. In addition, these findings suggested that the harmful activity of AgNPs-Cit-L-Cys might be correlated with their physicochemical properties and not solely attributed to the released Ag^+^ ions and confirmed that AgNPs-Cit-L-Cys phytoxicity is associated to oxidative stress.

## Introduction

Among various types of nanomaterials, silver nanoparticles (AgNPs) are the most commonly applied due to their physical and photochemical properties as well as their antibacterial and antifungal effects (Pokhrel et al. 2012; Cvjetko et al. 2018). AgNPs are utilized in many fields, including biomedical applications, diagnostics, environmental remediation, chemistry, food and textile industries (Ke et al. 2018; Fiorati et al. 2020; Ihtisham et al. 2021). In the field of agriculture, AgNPs have been applied to improve crop yield and as fungicides, pesticides, fertilizers, or fruit ripening agents (Yan and Chen 2019). Although AgNPs are not the most produced engineered nanomaterials, they result widely used in commercial products, so their discharge into the environment during all stages of their life cycle is definitively expected to significantly increase in the future, representing an emerging source of contamination (Peralta-Videa et al. 2011; Colman et al. 2013; Grün et al. 2018). As reported by Donner et al. (2015), the wastewater network has been identified as the major pathway for the release of AgNPs, allowing them to reach directly or indirectly the aquatic ecosystem, the soil and the atmosphere. In that regard, it has been estimated that most of produced AgNPs (around 17–22%) ends up in soils, mainly via land application of post-treated organic wastes (e.g., sewage sludge and biosolids) or wastewater effluent discharge, posing a potential threat to the ecosystems and consequently, human health (Cvjetko et al. 2018; Courtois et al. 2019). Several studies have demonstrated that AgNPs are toxic to bacteria, algae, terrestrial plants, human, and animal cells (Sung et al. 2008; AshaRani et al. 2009; Courtois et al. 2019; Behzadi Tayemeh et al. 2020; Bellingeri et al. 2022). Investigation on the potential phytotoxicity of AgNPs on plants has not yet be fully elucidated due to the influence of several variables on plant growth such as plant species, characteristics of the nanoparticles themselves (size, type, concentration, stability), experimental conditions and exposure time (Cox et al. 2017; Timoteo et al. 2019). However, at physiological and biochemical level, most publications report adverse effects of AgNPs, such as a decrease in plant growth, photosynthetic rate, chlorophyll content, and an increase in reactive oxygen species (ROS) production along with a significant induction in the activities of antioxidant enzymes (Yan and Chen 2019; Tortella et al. 2020; Ihtisham et al. 2021). It is still unclear whether the mechanisms of AgNPs toxicity are related to the release of silver ions (Ag^+^) or to the physical and chemical properties of the particles, including size, shape, and surface coating (Powers et al. 2011; Vishwakarma et al. 2017; Peharec Štefanić et al. 2018; Yan and Chen 2019). Consequently, it becomes very important to deepen our knowledge on the impact of AgNPs on terrestrial plants to properly regulate and safely dispose metal nanoparticles-based products and reduce their adverse impacts on environment. Hitherto, most of the studies concerning the phytotoxicity of AgNPs have been performed on food crops, annual herb, grass, and flowering plants (Budhani et al. 2019). Quite a few reports have investigated the effect of these nanoparticles on woody plants (Wang et al. 2013; Aleksandrowicz-Trzcińska et al. 2019; Cocozza et al. 2019). However, they have not focused on the interactions between AgNPs and poplar species under in vitro culture conditions upon chronic exposure. In vitro assay is considered a suitable method to evaluate stress responses in plants, especially in woody species characterized by long reproductive cycles (Confalonieri et al. 2003). Moreover, due to axenic and controlled conditions (light, temperature, humidity, and composition of nutrient media), plant cell culture reduces environmental variations that can affect the bioavailability of the toxic substances in the culture medium (Iori et al. 2012a; Ghorbanpour et al. 2018). Therefore, in the present study, we investigated the potential chronic toxicity of Ag in the form of bifunctionalized silver nanoparticles (AgNPs-Cit-L-Cys), novel nanomaterials intended for environmental applications in pollution monitoring and remediation (Schiesaro et al. 2020; Bellingeri et al. 2022; Iannelli et al. 2022), and silver nitrate (AgNO_3_) on callus culture of *Populus nigra* L. (clone Poli), a model plant used commonly in the research of abiotic stress, whose capability to tolerate environmental pollutants was previously assessed in in vitro approaches (Iori et al. 2012a, b).

## Materials and methods

### Synthesis and characterization of AgNPs-Cit-L-Cys

The AgNPs-Cit-L-Cys, used in this work, were synthesized using two highly hydrophilic capping agents, i.e., citric acid (Cit) and l-cysteine (l-Cys), in wet reduction reaction using the protocol described in previous works (Prosposito et al. 2019; Bertelà et al. 2021). The recipe followed for the synthesis is briefly reported here: l-Cys solution (25 mL, 0.02 M), Cit solution (10 mL, 0.01 M), and AgNO_3_ solution (2.5 mL, 0.05 M) were mixed in a flask and degassed with argon for 10 min. Then, sodium borohydride (NaBH_4_) solution (4 mL, 0.01 M) was added to the mixture. The reaction was carried out at room temperature for 2 h and the black product was recollected and purified by centrifugation. The obtained AgNPs-Cit-L-Cys were characterized, using Shimadzu 2401 PC UV–Vis spectrophotometer, showing the characteristic plasmonic peak at 420 nm due to their nanosize, consistent with previous studies for spherical shape particles (*Ø* < 10 nm) (Prosposito et al. 2016; Iannelli et al. 2022).

### Callus culture material and experimental setup

Callus cultures of *P. nigra* clone Poli were obtained by sub-culturing undifferentiated cell clusters from leaf tissue (Iori et al. 2012a). Calli were subcultured in Petri dishes on Murashige and Skoog (MS) nutrient medium (Murashige and Skoog 1962) supplemented with sucrose (30 g/L), kinetin (0.7 mg/L), 2,4-dichlorophenoxyacetic acid (2,4-D) (1 mg/L), and solidified with agar (7 g/L). The pH of the culture medium was adjusted to 5.7. Cultures were incubated in the dark at 25 °C in a growth chamber and subcultured every 3 weeks. The experimental treatment was conducted in callus culture conditions. After autoclaving and prior to solidification of the medium, either AgNPs-Cit-L-Cys or AgNO_3_ stock solutions were added to reach the following concentrations: 0, 2.5, 5 mg/L. For each treatment, five Petri dishes were used, each containing four calli. Petri dishes with callus culture without AgNPs or AgNO_3_ in the medium served as a control. Calli were exposed to treatment for 3 weeks. At the end of the exposure time, for the determination of fresh weight (FW) and dry weight (DW), each callus was collected, washed briefly with sterilized distilled water, dried on filter paper, and finally weighed. For biochemical analysis each callus was frozen in liquid N_2_ and stored in a freezer at − 80 °C.

### Determination of Ag content and bioconcentration factor

Calli were kept in an oven at 60 °C until constant weight was obtained. Then, the oven-dried material was weighed and mineralized. Mineralization was performed by treating 250 mg of dried samples with 9 mL of concentrated HNO_3_, 5 mL of distilled water and 1 mL of H_2_O_2_ (30% v/v in water), followed by heating (EXCEL Microwave Chemistry Workstation, Preekem Scientific Instruments Co., Ltd., Shanghai, China) in a four-step procedure: 100 °C for 1 min at 250 psi, 140 °C for 1 min at 350 psi, 170 °C for 1 min at 450 psi and 200 °C for 12 min at 550 psi. Samples were then filtered and analyzed. Determination of Ag concentrations was performed using Inductively Coupled Plasma Optical Emission Spectrometry (ICP-OES, 5800 Agilent Technologies, USA—LOD = 0.02 mg/L). The bioconcentration factor (BCF) was calculated to evaluate the ability of cell cultures to remove Ag (Iori et al. 2012a):

BCF = Ag concentration in the calli (mg/kg)/Ag concentration in the medium (mg/kg).

### Determination of malondialdehyde (MDA) content

The level of lipid peroxidation was determined by measuring malondialdehyde (MDA) content according to the modified protocol of Heath and Packer (1968). Briefly, frozen samples were homogenized in a pre-chilled mortar and pestle with two volumes of ice-cold 0.1% (w/v) trichloroacetic acid (TCA) and centrifuged for 15 min at 16,000 g. The reaction mixture containing 1 mL aliquot of supernatant and 2 mL of 0.5% (w/v) thiobarbituric acid (TBA) in 20% (w/v) TCA and 1 mM ethylenediamine tetraacetic acid (EDTA) was incubated at 95 °C for 30 min and then rapidly cooled in an ice-bath. The cooled mixtures were then centrifuged at 4 °C for 10 min at 16,000 g. The absorbance of the supernatant was measured at 532 nm by a spectrophotometer (Perkin Elmer, Norwalk, CT, USA) and values corresponding to non-specific absorption at 600 nm were subtracted. MDA concentration was calculated using the extinction coefficient (*ε* = 155 mM^−1^ cm^−1^).

### Assays of antioxidant enzymatic activities

To determine antioxidant enzyme activities, frozen samples were homogenized in a pre-chilled mortar and pestle with two volumes of an ice-cold 50 mM potassium phosphate buffer (pH 7.0) containing 0.1% (w/v) ascorbic acid, 1% (w/v) polyvinylpolypyrrolidone (PVPP), 1 mM Na_2_-EDTA, and 0.1% (v/v) Triton X-100. The homogenate was centrifuged at 4 °C for 30 min at 15,000 g and the supernatant fraction was set aside for assays of ascorbate peroxidase (APX) and catalase (CAT) activities. The total soluble protein content was quantified as described by Bradford (1976). Different aliquots of the supernatant fraction were mixed with 1 mL of Bradford dye reagent (Thermo Fisher Scientific), previously diluted with water 1:5 and the absorbance was monitored spectrophotometrically at 595 nm. The total soluble protein content in the supernatant fraction was calculated by comparison with bovine serum albumin (BSA) used as a standard.

Activity of ascorbate peroxidase (APX, EC 1.11.1.11) was determined by measuring the oxidation rate of ascorbate (*ε* = 2.8 mM^−1^ cm^−1^) at 290 nm for 1 min (Nakano and Asada 1981). Assay mixture contained 50 mM potassium phosphate buffer (pH 7.0), 0.5 mM ascorbic acid, 10 mM H_2_O_2_, and enzyme extract in a total volume of 1 mL. APX activity was expressed as µmol of ascorbate oxidized mg^−1^ protein min^−1^. Catalase (CAT, EC 1.11.1.6) activity was measured by following the consumption of hydrogen peroxide (*ε* = 36 mM^−1^ cm^−1^) at 240 nm for 40 s as described by Aebi (1984) with minor modifications. The reaction mixture was composed of 50 mM potassium phosphate buffer (pH 7.0), 125 mM H_2_O_2_, and enzyme extract in a total volume of 1 mL. CAT activity was expressed as µmol of H_2_O_2_ mg^−1^ protein min^−1^.

### Estimation of total phenolic content

About 1 g of frozen callus sample was homogenized with 10 mL of cooled 80% ethanol and placed on a shaker for 24 h in the dark at 25–30 °C. Then, the mixture was centrifuged at 4 °C for 25 min at 4000 g and the supernatant was collected for the quantification of total phenolics.

Total phenolic content was determined quantitatively using Folin-Ciocalteu (FC) reagent, with gallic acid as the standard by following the modified protocol of Bernabè-Antonio et al. (2021). Briefly, a diluted aliquot of ethanolic extract (1:10) was mixed with Na_2_CO_3_ (20% w/v) and FC in 1:1 (v/v) ratio. The mixture was incubated at room temperature for 60 min in the dark and its absorbance was measured spectrophotometrically at 760 nm against distilled water as blank. The total phenolic content was expressed as mg of gallic acid equivalents (GAE)/g of calli sample fresh weight (FW).

### Statistical analysis

All results were represented as mean of three replicates ± standard deviations (SD). Statistical analysis was performed using R software with one-way analysis of variance (ANOVA), followed by Tukey’s HSD post-hoc test. Significant differences compared to the control were obtained at *p* ≤ 0.05.

## Results

### AgNPs-Cit-L-Cys and AgNO_3 _effect on poplar calli growth

**Fig. 1:**
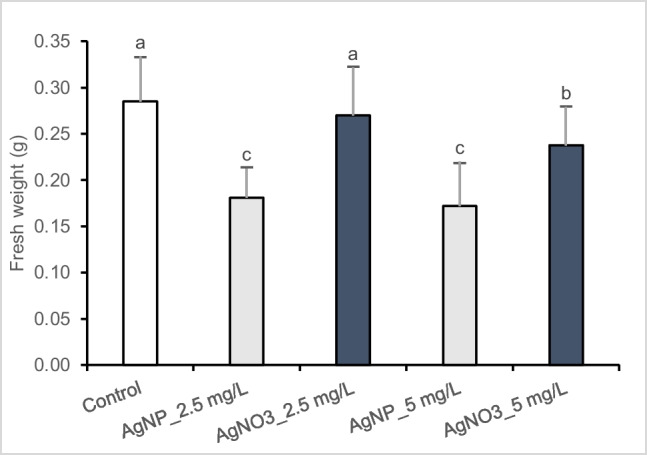
Fresh biomass production in callus cultures of *Populus nigra* L. clone Poli exposed for 3 weeks to different AgNPs-Cit-L-Cys and AgNO_3_ concentrations (± SD, *n* = 3). Different letters above bars indicate significant differences (*p* ≤ 0.05, Tukey’s test)

The effects of AgNPs-Cit-L-Cys and AgNO_3_ on the growth of *P*. *nigra* callus cultures were analyzed by evaluating biomass production (Fig. 1). The observed results indicated that AgNPs-Cit-L-Cys treatment had more harmful impact to poplar calli than AgNO_3_ when compared with control. Specifically, the exposure to 2.5 and 5 mg/L AgNPs-Cit-L-Cys, caused a reduction of 36.5% and 39.7%, respectively, in the fresh weight measurement of cell cultures with respect to the control whereas, in the presence of 2.5 and 5 mg/L AgNO_3_ the reduction was of 5.3% and 16.5%, respectively.

### Protein content

**Fig. 2:**
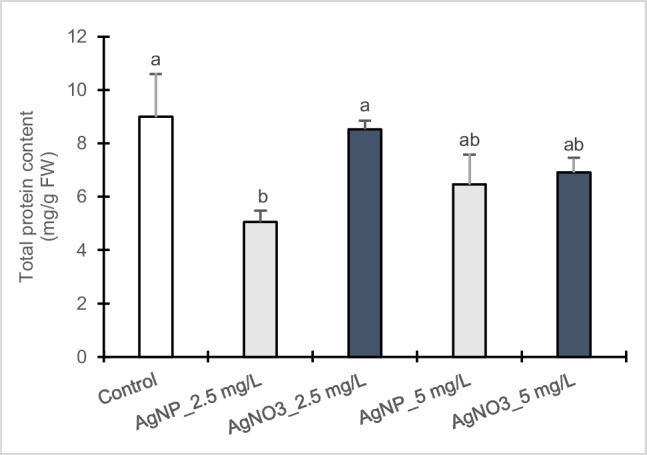
Total protein content in callus cultures of *Populus nigra* L. clone Poli exposed for 3 weeks to different AgNPs-Cit-L-Cys and AgNO_3_ concentrations (± SD, *n* = 3). Different letters above bars indicate significant differences (*p* ≤ 0.05, Tukey’s test)

The effects of AgNPs-Cit-L-Cys and AgNO_3_ on the total soluble protein content were evaluated on poplar callus cultures (Fig. 2). In particular, at 2.5 and 5 mg/L AgNPs-Cit-L-Cys results showed a reduction of 50% and 35% in total protein content, respectively, compared to control, whereas at the corresponding AgNO_3_ concentrations a lower reduction was observed (14% and 30%, respectively), suggesting that AgNPs-Cit-L-Cys had a more toxic effect than AgNO_3_.

### Oxidative damage determination by lipid peroxidation

**Fig. 3:**
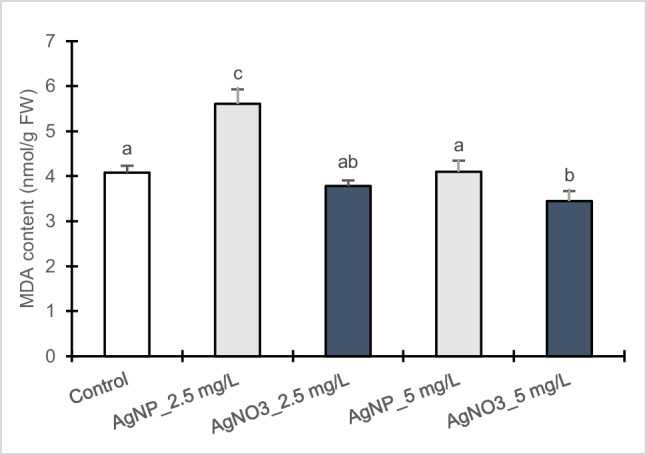
Lipid peroxidation content (MDA) in callus cultures of *Populus nigra* L. clone Poli exposed for 3 weeks to different AgNPs-Cit-L-Cys and AgNO_3_ concentrations (± SD, *n* = 3). Different letters above bars indicate significant differences (*p* ≤ 0.05, Tukey’s test)

As reported in Fig. 3, a significant increase in lipid peroxidation level, through MDA measurement, was detected in poplar callus cultures exposed to 2.5 mg/L AgNPs-Cit-L-Cys (27.36%) compared to control, whereas at 5 mg/L no statistical differences were observed. Instead, for calli exposed to AgNO_3_ treatment, a slight significantly decrease was found at 5 mg/L (15.6%), whereas at 2.5 mg/L the values were not statistically different with respect to control. Furthermore, between the bifunctionalized AgNPs-Cit-L-Cys and AgNO_3_ treatments, significant differences were detected in MDA level, especially at 2.5 mg/L, highlighting more damaging effects of AgNPs-Cit-L-Cys respect to AgNO_3_.

### Antioxidant enzymatic activities

**Fig. 4:**
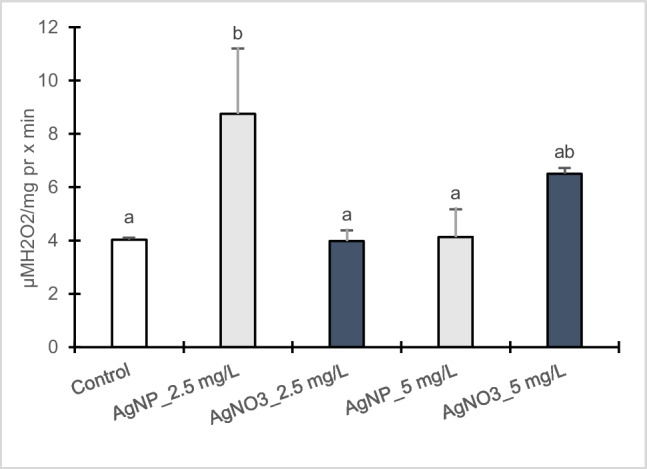
Catalase activity in callus cultures of *Populus nigra* L. clone Poli exposed for 3 weeks to different AgNPs-Cit-L-Cys and AgNO_3_ concentrations (± SD, *n* = 3). Different letters above bars indicate significant differences (*p* ≤ 0.05, Tukey’s test)

**Fig. 5:**
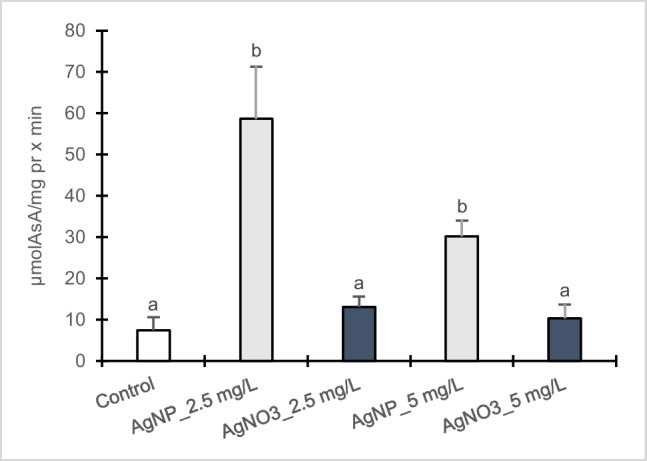
Ascorbate peroxidase activity in callus cultures of *Populus nigra* L. clone Poli exposed for 3 weeks to different AgNPs-Cit-L-Cys and AgNO_3_ concentrations (± SD, *n* = 3). Different letters above bars indicate significant differences (*p* ≤ 0.05, Tukey’s test)

The outcomes related to enzymatic antioxidants such as CAT and APX are presented in Figs. 4 and 5. Poplar callus cultures showed a significant enhancement in CAT activity when exposed to 2.5 mg/L AgNPs-Cit-L-Cys whereas at 5 mg/L no statistical difference was observed compared to control. On the contrary, CAT activity increased in dose-dependent manner in the presence of AgNO_3_, resulting 61% higher at 5 mg/L than at 2.5 mg/L with respect to control. Between AgNPs-Cit-L-Cys and AgNO_3_ treatments, significant differences were observed. In particular, at 2.5 mg/L, a greater increase in CAT activity was found in poplar calli exposed to AgNPs-Cit-L-Cys compared to the corresponding AgNO_3_ treatment. Conversely, a higher CAT activity was observed in callus cultures exposed to 5 mg/L AgNO_3_ compared to the corresponding AgNPs-Cit-L-Cys treatment.

APX enzymatic activity increased in both treatments with respect to control, resulting more evident in poplar callus cultures exposed to AgNPs-Cit-L-Cys than to AgNO_3_. In the presence of AgNPs-Cit-L-Cys, APX activity resulted significantly higher at 2.5 mg/L than 5 mg/L, compared to control. On the contrary, upon AgNO_3_ treatment no statistical differences were found in APX activity at both 2.5 and 5 mg/L.

### Total phenolic content

**Fig. 6:**
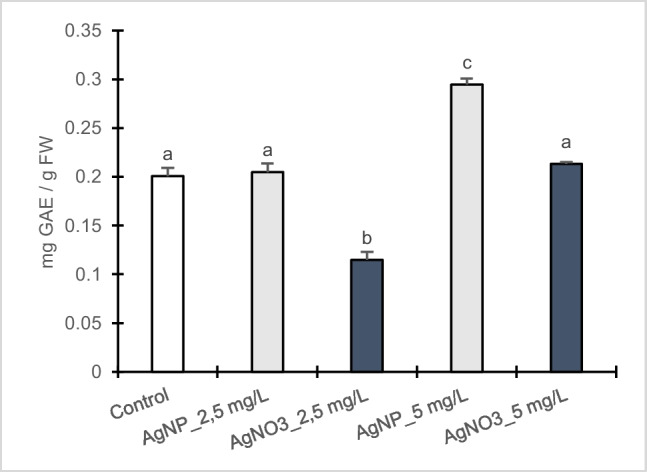
Total phenolic content in callus cultures of *Populus nigra* L. clone Poli exposed for 3 weeks to different AgNPs-Cit-L-Cys and AgNO_3_ concentrations (± SD, *n* = 3). Different letters above bars indicate significant differences (*p* ≤ 0.05, Tukey’s test)

Total phenolic amount increased in dose-dependent manner in both treatments (Fig. 6). In particular, poplar calli showed a significant enhancement in the content of phenols when exposed to 5 mg/L AgNPs-Cit-L-Cys, whereas at 2.5 mg/L no statistical difference was observed compared to control. In AgNO_3_ treated calli, total phenolic content increased significantly at 5 mg/L compared to the lowest concentration but the values were not statistically different with respect to control.

### Total Ag determination and bioconcentration factor

**Fig. 7:**
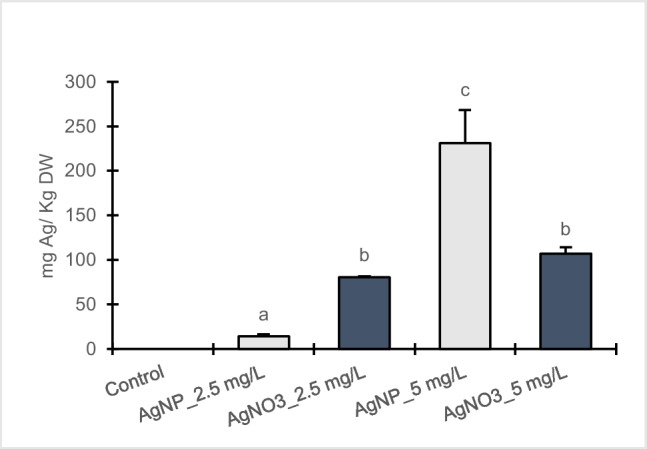
Ag^+^ concentration in callus cultures of *Populus nigra* L. clone Poli exposed for 3 weeks to different AgNPs-Cit-L-Cys and AgNO_3_ concentrations (± SD, *n* = 3). Different letters above bars indicate significant differences (*p* ≤ 0.05, Tukey’s test)

In poplar callus cultures exposed to either AgNPs-Cit-L-Cys or AgNO_3_, silver accumulation increased significantly in dose-dependent manner compared to control (Fig. 7). Between the applied AgNPs-Cit-L-Cys and AgNO_3_ treatments, highly significant differences were observed. At 2.5 mg/L, a noteworthy increase in Ag accumulation was detected in calli exposed to AgNO_3_ compared to the corresponding AgNPs-Cit-L-Cys treatment. On the contrary, a higher Ag concentration was found in calli exposed to 5 mg/L AgNPs-Cit-L-Cys compared to the corresponding AgNO_3_ treatment.

**Fig. 8:**
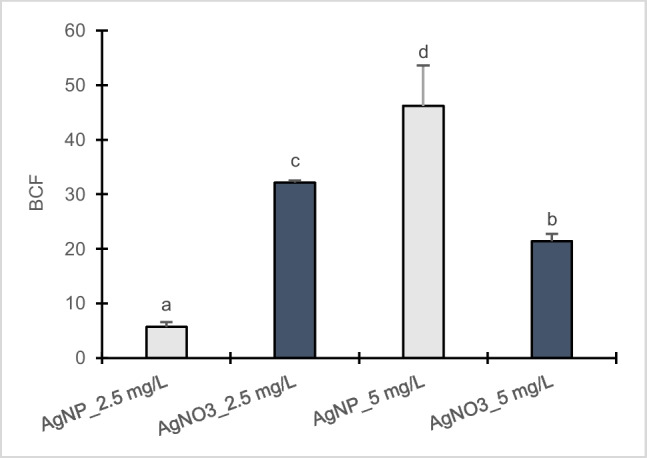
Bioconcentration factor (BCF) in callus cultures of *Populus nigra* L. clone Poli exposed for 3 weeks to different AgNPs-Cit-L-Cys and AgNO_3_ concentrations (± SD, *n* = 3). Different letters above bars indicate significant differences (*p* ≤ 0.05, Tukey’s test)

As reported in Fig. 8, in calli of clone Poli BCF value resulted significantly elevated at 5 mg/L AgNPs-Cit-L-Cys compared to 2.5 mg/L whereas an opposite effect was observed after AgNO_3_ exposure.

## Discussion

Despite the predicted environmental concentration of AgNPs ranges between 0.03 and 0.08 mg/L (Ihtisham et al. 2021), in the current work higher AgNPs-Cit-L-Cys concentrations as well as longer exposure time were chosen in order to induce a detectable response in plant cells and to assess the potential NPs environmental risk. The chronic toxicity was evaluated by analyzing biochemical and physiological parameters associated to stress response.

A commonly used physiological parameter for assessing phytotoxicity of AgNPs is the biomass production. The published literature is quite contradictory, as inhibitory or stimulatory effects were both reported, being correlated to plant species, nanoparticles type, size and concentration, and the period of exposure. For instance, it has been shown that AgNPs at 50 mg/mL increased significantly the mean callus fresh weight of *Phaseolus vulgaris* L. after 30 min exposure (Mustafa et al. 2017). Similarly, AgNPs at 40 and 60 mg/L caused an enhancement in biomass accumulation in callus cultures of sugarcane (*Saccharum* spp.) (Iqbal et al. 2019). Contrarily, it has been observed that fresh and dry mass of pearl millet (*Pennisetum glaucum* L.) seedlings reduced significantly after 24 h exposure to increasing concentration of AgNPs (Khan et al. 2019). Furthermore, *Lemna* plants exposed to AgNPs-Cit-L-Cys at both 20 and 50 mg/L for 14 days showed a biomass increase in respect to control (Iannelli et al. 2022).

Overall, the majority of studies reported a greater detrimental impact of AgNO_3_ on plant growth in respect to AgNPs. On the contrary, in the current research a major toxic effect on poplar fresh mass was observed after AgNPs-Cit-L-Cys addition to the culture medium compared to AgNO_3_. Although the mechanism of phytotoxic action of AgNPs is still unclear and not fully described, it is conceivable that AgNPs-Cit-L-Cys toxicity cannot be ascribed merely to the activity of the released Ag^+^ ions, known to be readily bioavailable, but it can be correlated with the intrinsic properties of the nanoparticles, as reported by Yan and Chen (2019). Particularly, several studies showed that smaller AgNPs (≤ 20 nm), as the NPs used in the current study, have stronger impact on plant growth than larger AgNPs (≥ 20 nm) due to their higher surface areas and, hence, they can better interfere with cell membrane function, affecting its permeability and, consequently, water and nutrient uptake (Ma et al. 2010; Navarro et al. 2008; Yin et al. 2011; Cvjetko et al. 2018).

The decrease in biomass production could be correlated with the oxidative stress induced by silver nanoparticles. Many studies have established that AgNP exposure can generate excessive amounts of reactive oxygen species (ROS), leading to irreversible damage on lipids, DNA and proteins and resulting in growth inhibition (Ma et al. 2015; Yan and Chen 2019). In this study, total protein content decreased significantly in both treatments compared to control and a greater reduction was observed after the exposure to AgNPs-Cit-L-Cys than AgNO_3_. In general, these outcomes are consistent with Al-Huqail et al. (2018) who observed a significant reduction in protein content after a 10-day exposure of *Lupinus termis* seedlings to high concentrations of AgNPs (300 and 500 mg/L). As mentioned by McShan et al. (2014), an important mechanism of toxicity for AgNPs is the interaction of both nanoAg^0^ and Ag^+^, with proteins and amino acids due to the strong affinity of silver for sulfur, causing protein conformational changes or even protein damage.

In addition, changes in ROS generation can cause peroxidation of polyunsaturated fatty acids (known as lipid peroxidation) (Ma et al. 2015; Tripathi et al. 2017). Malondialdehyde (MDA) is a major peroxidation product and is considered as biomarker of the extent of lipid peroxidation (Yan and Chen 2019; Iannelli et al. 2022). Our results showed that in poplar calli, AgNPs-Cit-L-Cys treatment induced an increase in MDA level, especially at 2.5 mg/L, while no lipid peroxidation was observed in the presence of AgNO_3_. These results are in accordance with those reported for wheat callus (Barbasz et al. 2016), rice (*Oryza sativa* L.) (Shaw and Hossain 2013), potato (*Solanum tuberosum* L.) (Homaee and Ehsanpour 2015), and mung bean (*Vigna radiata* L.) (Nair and Chung 2015) treated with AgNPs where an enhancement in lipid peroxidation was observed, confirming that in Poli calli toxic effect of AgNPs-Cit-L-Cys is associated to oxidative stress.

To counteract the detrimental effects of excessive production of ROS induced by AgNPs, plant cells activate a defense mechanism involving several antioxidative enzymes (e.g., CAT and APX) and metabolites (e.g., phenols, flavonoids, and anthocyanins). In calli of clone Poli, the lowest AgNPs-Cit-L-Cys concentration induced higher CAT and APX activities with respect to control. Contrary, no changes were observed in APX activity after AgNO_3_ treatment whereas CAT activity increased in dose-dependent manner, resulting higher at 5 mg/L compared to control. A similar trend in the induction of CAT and APX activities under AgNPs-Cit-L-Cys treatment was observed in two different previous studies in which Poli calli were exposed to Cd (Iori et al. 2012a) and ibuprofen (Iori et al. 2012b). In particular, in both studies a significant increase in CAT and APX activities was detected at the lowest pollutant concentration, highlighting a very sensitive callus culture response in order to counteract the effects of the xenobiotics. As evidenced by Khan et al. (2019) the impact of AgNPs on the antioxidant enzymes varied with plant species, their concentration and type, exposure medium, and treatment time. Consequently, comparison across separate studies may result confounded. Jiang et al. (2014) reported an increase in CAT activity in *Spirodela polyrhiza* L. exposed to AgNPs. Similarly, potato plantlets (*Solanum tuberosum* L.) showed an increase in CAT and APX upon AgNPs treatment (Homaee and Ehsanpour 2016). An enhancement in APX and CAT activities was also found in *Lemna* species in respect to different AgNPs-Cit-L-Cys concentrations as well as prolonged exposure time (Iannelli et al. 2022). On the contrary, Cvjetko et al. (2017) observed a decrease in CAT and APX activities in *Allium cepa* L. roots treated with AgNPs.

An enhancement in antioxidant activity might be correlated to an increase in the production of secondary metabolites, including phenols, with antioxidative properties and capable of directly scavenging free radicals (Sharma et al. 2019). Phenolic compounds have been reported to support the antioxidant enzyme system to remove and repair ROS-induced damage and can interact with other substances, such as vitamins C/E and carotenoids, producing synergistic antioxidant effects (Lv et al. 2021). In poplar calli, an increase in total phenolic content was noted at the highest AgNPs-Cit-L-Cys concentration. Interestingly, in AgNO_3_ treated calli total phenolic content increased in dose-dependent manner, resulting higher at 5 mg/L but not significantly different compared to control.

Several studies have been performed to investigate the potential role of nanoparticles as abiotic elicitors in many plants such as *Solanum tuberosum* L. (Homaee and Ehsanpour 2016), *Caralluma tuberculata* (Ali et al. 2019), *Cucumis Anguria* L. (Chung et al. 2018), and *Lavandula angustifolia* Mill. (Jadczak et al. 2020), showing that high concentrations of AgNPs induced an increase in phenolic compounds content. The mechanism of action through which nanoparticles elicit secondary metabolism is not yet determined. As reported by Ali et al. (2019), nanoparticles might act as signal compounds and affect the production as well as the composition of secondary metabolites involved in the antioxidant defense mechanisms.

Overall, based on the obtained results on oxidative stress parameters, AgNPs-Cit-L-Cys exhibited an increased toxic impact at 2.5 mg/L than at 5 mg/L in calli of clone Poli. Interestingly, this effect is not connected with higher Ag uptake as at 2.5 mg/L AgNPs-Cit-L-Cys, poplar calli accumulated less silver than at 5 mg/L, resulting in a lower BCF value.

Taking into account that the mechanism of action of AgNPs is still unknown, it is likely that in poplar calli the two different responses induced at cellular level in the presence of 2.5 and 5 mg/L AgNPs-Cit-L-Cys could be related to the phenomenon of hormesis. As reported by Bell et al. (2014), hormesis is the nonlinear dose response relationship in which a low and high dose of a chemical compound or an environmental factor can cause effects in opposite directions. Some types of NPs exhibit toxicity at low doses, depending on their intrinsic properties, such as specific sizes, shapes, and surface charges as well as the environmental medium in which they interact. As evidenced by Rascol et al. (2016), nanoparticles-cell membranes interactions represent a critical step, underlying the NP cytotoxicity. In this regard, it is conceivable that at 2.5 mg/L, AgNPs-Cit-L-Cys were able to keep their toxic potential and, upon contact with cell membrane, promoted hole, or pore formation in the lipid bilayer, leading to the impairment of cell wall functionality and to significant changes in the metabolism of Poli calli, resulting in a decrease in Ag accumulation as well as a lower value of BCF. Likewise, Torrent et al. (2020) found that *Lactuca sativa* L. exposed to PVP-AgNPs could not accumulate more silver in roots as they started to suffer detrimental effects and their metabolism was affected. On the contrary, it is likely that at 5 mg/L, AgNPs-Cit-L-Cys underwent somehow to modifications that quenched the originally hyper-reactive surface, resulting in a mild toxic effect, as observed by Cvjetko et al. (2018) in tobacco plants exposed to AgNPs coated with citrate. Consequently, despite of the increase in Ag accumulation and the higher BCF value detected, Poli calli maintained the ability to control silver nanoparticles influx and efflux properties, possibly by APX activity and antioxidant action of phenolic compounds, able to inhibit lipid peroxidation and hamper the diffusion of free radicals.

## Conclusion

To our knowledge, this is the first work showing the effects of the bifunctionalized silver nanoparticles (AgNPs-Cit-L-Cys) and silver nitrate (AgNO_3_) on a woody plant species, *Populus nigra* L. (clone Poli), in an in vitro assay upon chronic exposure. Between the two treatments conditions, our findings showed that AgNPs-Cit-L-Cys caused major detrimental effects on poplar callus cultures, resulting more evident at lower concentration (2.5 mg/L). Interestingly, at this concentration, the presence of AgNPs-Cit-L-Cys induced a remarkable decrease in fresh biomass of poplar calli, a greater MDA content and a notable enhancement of APX and CAT activities, despite the lower Ag accumulation as well as BCF value. These results confirm that phytotoxicity of AgNPs-Cit-L-Cys is associated with oxidative stress, leading to the activation of defense mechanisms, involving antioxidative enzymes. In this regard, further research is necessary for a better understanding of AgNPs-Cit-L-Cys uptake, accumulation, and their impact on secondary metabolism also at molecular level, in the perspective of a safer environmental application.

## Data Availability

The datasets in this study are available from the corresponding author upon reasonable request.
